# Do obesity-promoting food environments cluster around socially disadvantaged schools in Glasgow, Scotland?

**DOI:** 10.1016/j.healthplace.2012.06.001

**Published:** 2012-11

**Authors:** Anne Ellaway, Laura Macdonald, Karen Lamb, Lukar Thornton, Peter Day, Jamie Pearce

**Affiliations:** a MRC Social and Public Health Sciences Unit, Glasgow, UK; b Deakin University, Australia; c University of Canterbury, New Zealand; d University of Edinburgh, UK

**Keywords:** Food outlets, Schools, Social disadvantage, Obesity

## Abstract

Increase in the consumption of food and drinks outside the home by adolescents and young people and associations with rising levels of obesity is a significant concern worldwide and it has been suggested that the food environment around schools may be a contributory factor. As few studies have explored this issue in a UK setting, we examined whether different types of food outlets are clustered around public secondary schools in Glasgow, and whether this pattern differed by social disadvantage. We found evidence of clustering of food outlets around schools but a more complex picture in relation to deprivation was observed. Across all schools there were numerous opportunities for pupils to purchase energy dense foods locally and the implications for policy are discussed.

## 1 Background

In recent years there has been a large increase in the consumption of drinks and foods outside of the home by adolescents and young people (Lachat et al., 2012), with associated concerns over the energy dense nature of these foods and links with obesity (Prentice and Jebb, 2003). Growing levels of obesity and associated health problems among children and young people is a major concern worldwide. Scotland has one of the highest levels of obesity in OECD countries, with only the USA and Mexico exceeding these rates (Donnelley et al., 2010). In Scotland, around a third of 11 year olds are overweight or obese, with higher rates in deprived areas (Corbett et al., 2010). Obesity in childhood has been linked to a range of poorer health outcomes in adulthood (Lobstein et al., 2004) and understanding the potential of the local social and physical environment to influence modifiable determinants of obesity is seen as crucial (Egger and Swinburn, 1997). Although the causes of obesity are complex and multi-factorial, the role of food retail environments around schools has been mooted as a potential determinant of obesity, as young people (particularly those in secondary schools) have greater freedom to purchase foods independently (NICE, 2010). Concerns over the consumption of unhealthy foods, ‘fast foods’ in particular, has resulted in calls to develop local planning guidelines in some areas in the UK to restrict the siting and licensing of fast food outlets near schools (NICE, 2010). A small number of studies (mainly North American or New Zealand) have found fast food outlets to be located close to schools, particularly those in poor areas (Austin et al., 2005; Davis and Carpenter, 2009; Day and Pearce, 2011; Simon et al., 2008; Sturm, 2008), however to our knowledge few studies have examined this in a UK setting (an exception is a study of one London borough (Lloyd et al., 2010)).

In this study therefore we evaluate whether different types of food outlets cluster around secondary schools in Glasgow, Scotland, and whether the degree of clustering of different types of outlets is greater around schools with a high proportion of students from socially disadvantaged backgrounds. We focus our study on secondary schools as primary school pupils are much less likely to be permitted to leave school premises at lunchtime to purchase food.

## 2 Methods

### 2.1 Data

A database of food retailers was created (as at July 2011) using a list of food retailers held by Glasgow City Council (GCC). The list of food retailers (*n*=2236) was held by the Council for licensing, inspection and planning purposes, and included all premises that fall under the 1995 Food Safety and Hygiene guidelines (DEPARTMENT OF HEALTH, 1995). Although there are a range of methods to classify retail outlets (such as The Town and Country Planning (Use Classes) Order 1987 categories in the UK), we used the Council defined categories, which included ‘cafes’, ‘takeaways’, ‘food stores’ (e.g. general stores, newsagents, supermarkets), ‘multi-national fast food chains’ and fixed stance food vans. The accuracy of the list was checked using the on-line Yellow Pages and the websites of multi-national fast food chains (e.g. McDonald's). Whilst the use of ‘off the shelf’ local authority food outlet data to characterise the local ‘foodscape’ is not ideal (Lake et al., 2012) it has been shown to be reasonably accurate, particularly in urban areas (Cummins and Macintyre, 2009; Lake et al., 2010, 2012).

All public secondary schools in Glasgow (*n*=29) and all food retailers were mapped using street maps (including point addresses) obtained from UK Ordnance Survey (ORDNANCE SURVEY, 2010) and a Geographical Information System (GIS) was used to geocode schools and food retailers by their unit postcodes. A unit postcode is the lowest level of postal geography used in the UK and contains, on average, 16 households/premises. GIS was used to create buffer zones, with straight-line boundaries of 400 and 800 m around each school, and a count of food retailers was calculated for each school. We chose to use straight-line boundaries as road network distances may not adequately capture the various routes that young people may take when they exit their school, for example, they may use alleyways, back lanes or small paths around their school. That said, in our study the distances measured by the straight-line and road network methods of creating buffer zones were highly correlated (a correlation of 0.88 for the 400 m distance and 0.84 for the 800 m distance). Distances of 400 m and 800 m were selected to approximate a 5 and 10 min walk, respectively (Austin et al., 2005; Day and Pearce,. 2011). Although we were particularly interested in 400 and 800 m distances, we also examined distances of 1200 and 1500 m from the school locations, as adopted in other studies (Austin et al., 2005; Day and Pearce,. 2011) as some pupils may have the opportunity to walk further at lunchtime, as well as pass food outlets on their way to and from school.

The percentage of pupils receiving free school meals (a benefit based on low household income) was used to classify the schools by deprivation and grouped into 4 quartiles from least deprived (quartile 1, average of 16% of pupils eligible for free school meals) to most deprived (quartile 4, average of 41% eligible for free school meals) (Learning and Teaching Scotland, 2010). Schools were classified by this measure of household poverty rather than the area level deprivation index of each school as a school's location may not necessarily coincide with the socio-demographic profile of its pupils, given that secondary school pupils come from a wider catchment area than the area immediately surrounding the school. It should be noted that the population of Glasgow is poor compared to the rest of Scotland, with the average amount of pupils (29%) eligible for free school meals around twice that of the Scottish average (14%).

## 3 Statistical analysis

To determine whether there is evidence of spatial clustering of food outlets around schools, spatial point pattern analysis using a bivariate *K*-function was adopted. The calculated *K*-function is defined as the expected number of points of type *j*, i.e. food outlets, within a distance *r* of a point of type *i*, i.e. schools, divided by the average number of points of type *j* in the area (Baddeley, 2008). Under complete spatial randomness (CSR), the *K*-function has a simple form: *K=πr*^*2*^. The ratio of the observed *K*-function curve was divided by the expected *K*-function curve in order to assess the clustering at the predefined distance thresholds from the schools. A ratio of one indicates no statistical evidence of clustering, with the observed pattern of food outlets around schools showing no difference from a random pattern. A ratio higher than one indicates evidence of clustering. Simulation envelopes based on 100 simulations under the assumption of no spatial dependence were created around the expected *K*-function curve to examine the statistical significance of the clustering. These simulation envelopes are critical values in a test of the hypothesis of no clustering in the data. If the observed *K*-function appears above the plotted simulation envelope there is evidence of significant clustering.

The clustering of all outlets around schools was considered and the analysis was also stratified by outlet type as well as by school deprivation quartile. The benefit of this approach is that it allows an examination of the degree of clustering at different distances from the schools by providing an estimate of the measure of spatial dependence. In order to adjust the *K*-function for secondary schools located at the edge of the study area, Ripley's edge correction method was adopted (Ripley, 1988). This correction was applied as it is not possible to observe points of the random pattern outside the study region.

All descriptive analysis was carried out using SPSS version 15.0. The spatial analysis was carried out using ArcGIS version 9.1 and the Spatstat package in R version 2.13.1 (Baddeley and Turner, 2005).

## 4 Results

**Fig. 1:**
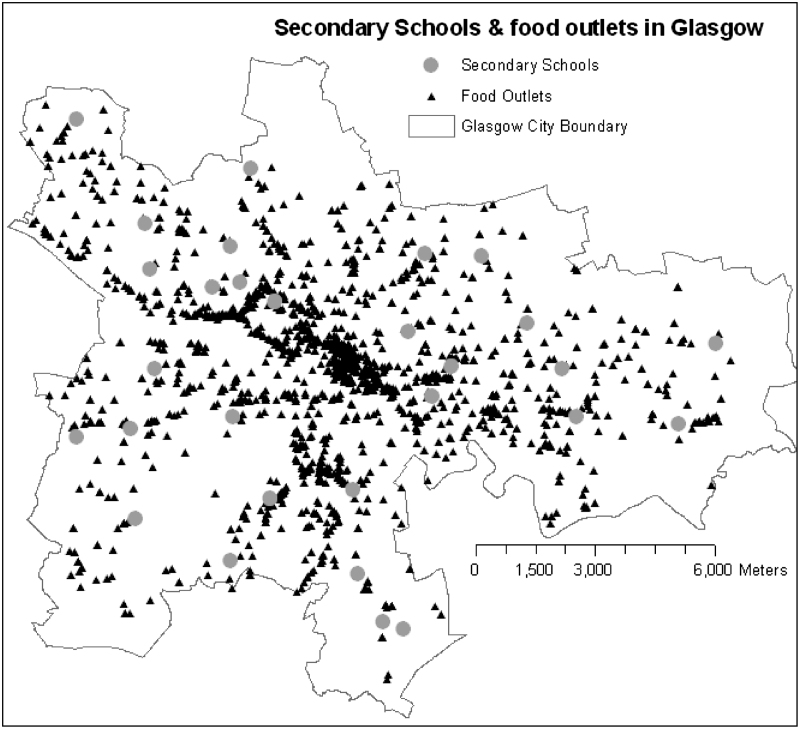
Location of secondary schools and food outlets in Glasgow.

Fig. 1 shows the location of all food outlets and secondary schools included in the analysis. The types of food outlets are described in Table 1. The most common type of food outlet in Glasgow is the takeaway, accounting for 39.0% (*n*=873) of all food outlets, followed by food stores (33% of all outlets; *n*=735) whilst multi-national fast food chains are relatively rare, representing only 1.5% (*n*=33) of all food outlets. Of the 2236 food outlets examined, only 12% of them (271 outlets) were sited within a 5 min walk (400 m) of secondary schools across Glasgow. Of these outlets, over a third were located in the least deprived quartile of schools. However, at a 10 min walk (800 m), almost half of the outlets (*n*=1007) were located within this distance, providing an average of 35 opportunities per school (median =24; range 4–100) with schools in quartile 3 (second most deprived) having the highest proportion of outlets (328 outlets; median =60, range 7–91).

**Fig. 2:**
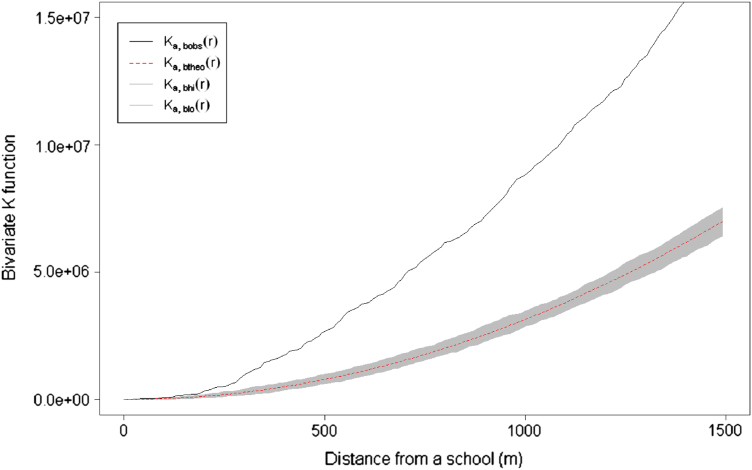
Bivariate *K*-function plot with pointwise simulation envelopes showing clustering of all fast food outlets around schools.

Fig. 2, there was evidence of clustering of food retail outlets around schools at all distances examined with the exception of national fast food chains (apart from weakly at 1500 m) (Table 2). When we examined this by deprivation, we found that clustering (at 400 m) for all food outlets was highest around schools in the more affluent quartile (Q1) followed by the second most deprived quartile (Q3). At 800 m distance, again the second most deprived quartile (Q3) showed the highest level of clustering.

The clustering of different food outlet types around schools by deprivation quartile was also examined (Table 3). Cafes, takeaways and food stores were more highly clustered around schools in the most affluent quartile using the 400 m distance measure; whereas at a ten minute walk distance (800 m), evidence for clustering of these three types of outlet was strongest in the second most deprived quartile. There was little evidence of clustering of national fast food chain outlets around any of the school quartiles at any of the four distances examined, most of the potential for the purchase of energy dense foods by pupils seems to emanate from takeaways and food stores nearby.

## 5 Discussion

An important finding from this research is that there is a high level of locational access to food stores around schools in Glasgow. This observation is consistent with other studies from a range of countries that have examined the ‘obesogenic’ characteristics of environments around schools. However, contrary to the evidence from elsewhere (e.g. North America, New Zealand), we have found no clear pattern of clustering of food outlets per se or by socio-economic deprivation around Glasgow secondary schools. Evidence for clustering appears to be dependent on the type of food outlet and the distance measure examined. Cafes and takeaways tended to cluster within a five minute walk (400 m) of schools with fewer pupils from poorer households (bearing in mind the high levels of deprivation in Glasgow compared to the rest of Scotland). These particular schools tend to be located closer to established high streets and arterial roads. However, the clustering of food outlets near these schools has the potential to reach greater numbers of pupils as these schools have approximately twice the number of pupils compared to schools in more deprived areas. Some of these larger schools are faith based and draw pupils from a wide catchment area, others have a broad socio-economic and ethnic composition. When we looked at longer distances (800 m), evidence for clustering for food stores, takeaways and cafes was higher in schools in the second most deprived quartile.

We found no evidence that the major international fast food chains are located within easy reach of Glasgow school children at lunchtime, although access may differ by pupils' journey to and from school. Although the foods consumed by pupils on their journey to and from school may make a significant contribution to energy intake among pupils (Borradaile et al., 2009), we do not have any extensive information on the routes or mode of travel that pupils take (however a recent survey across a range of West of Scotland schools showed that less than 50% of secondary school pupils walked to school and that this figure was higher among pupils who lived closer to school and from more deprived backgrounds (Sustrans, 2011)). We also found few vans per school, however, these data were based on information provided by Glasgow City Council on the number of licensed vans, anecdotal evidence suggests that unlicensed vans are driving to schools and selling foods to pupils at lunchtime. Most of the opportunities for pupils to purchase energy dense foods appear to emanate from local takeaways and food stores as time constraints on pupils at lunchtime would not allow sit-down meals in cafes (although some will have takeaway counters).

The limitations of our study are that it is based in one densely populated Scottish city and that exploring this issue in a range of areas (e.g. suburbs) may have given different results. Our exposure measure was based on buffer zones using a single geocoded point for the school, and does not take into account different entry/exit points that pupils may commonly use or the road network that pupils would traverse to access food outlets. The *K*-function assumes a planar space of straight line distance between points which could lead to the over detection of clustered patterns in road network constrained food outlet points (Okabe and Yamada, 2001). We do not know which food outlets are popular with pupils, the types of foods they purchase and whether food outlets are open at lunchtime or are promoting particular foods to pupils (this is the subject of another study currently underway). However, a strength of our study is that we examined a range of food outlets around schools, including small local food shops such as convenience or corner stores often absent from discussion of the food retail environment around schools (Howard et al., 2011; Neckerman et al., 2010).

There are a range of complex and inter-related factors that might explain the distribution of food outlets in proximity to schools, including urban design, historical factors, planning regulation, co-location of other businesses and land prices (Pearce and Day, 2010). Demand and supply side factors (both current and historical) need to be taken into account, decisions of where to locate and what to sell are likely to be informed by the type of customer base located in particular areas and what they are likely to buy as well as the opportunity afforded to maximise sales by being located along main arterial roads (Melaniphy, 2007).

This study has demonstrated that in Glasgow there are on average 35 food outlets within a ten minute walk of secondary schools (with the degree of exposure varying across city schools). At present, the environment around Glasgow schools provides ample opportunities to purchase food options. Such environmental exposures might hamper efforts to improve dietary behaviours amongst adolescents given these exposures would interact with other factors (e.g. social norms, peer pressure) that encourage the purchasing and consumption of energy dense foods. Similarly, further research could usefully consider the positioning of food outlets along the routes taken by students to and from school, as well as the policies in place within schools that promote healthy eating (Walton et al., 2009). Some Glasgow secondary schools have implemented a ‘stay-on-site’ policy to encourage pupils to purchase lunch from school canteens which only sell foods which meet the minimum nutritional standards that are set for food consumption on school premises (The Scottish Goverment, 2008). The extent to which any ‘stay-on-site’ policy is enforced, the canteen environment, staggered lunch times (to reduce queues and overcrowding) and what other lunch-time activities (such as clubs) are in place to occupy pupils have all been shown to be associated with an increased uptake of school meals, increased pupil safety, better relations within the school between staff and pupils, and pupils settling in more quickly to secondary school (Beaulieu and Godin, 2011; Scottish centre for social research, 2011). However, given the continuing popularity among pupils of purchasing lunchtime foods off-site (only a third of Glasgow's secondary pupils currently eat a school lunch (The Scottish Government 2010, 2010)), more attention needs to be given to the food retail environment around secondary schools in Glasgow. A better understanding of the push and pull factors driving the lunchtime exodus from school is warranted, including price factors for particular foods and drinks (e.g. one US study found that it was price rather than availability that determined the level of consumption of energy dense foods among adolescents (Powell, 2009)); while another US study of adolescents found greater price sensitivity for sugar-sweetened carbonated drinks than for burgers (Gordon-larsen et al., 2011). The consumption of confectionery and sugar-sweetened carbonated drinks by Scottish teenagers is amongst the highest in Europe (Mcneill et al., 2010; Currie et al., 2007) and may be a pull factor as they are not available to buy within school premises but are freely available to purchase off-site.

Our findings provide support for the development of interventions and policies which discourage new outlets selling energy dense foods, working with local shopkeepers to encourage them to stock healthier alternatives at competitive pricing (Andreyeva et al., 2011) as well as individual and school level interventions.

## Figures and Tables

**Table 1:** Number (and %) of each food outlet type in Glasgow; number (and %) within 400 m and within 800 m of schools by deprivation quartile of school⁎.

		**Within 400** **m**					**Within 800** **m**				
**Food outlet type**	**N**										
	**(%) of total outlets across Glasgow**	**All**	**Q1**	**Q2**	**Q3**	**Q4**	**All**	**Q1**	**Q2**	**Q3**	**Q4**

	N (%)	N (%)									
			N (%)	N (%)	N (%)	N (%)	N (%)	N (%)	N (%)	N (%)	N (%)
**Cafés**	436	45	19 (42.2)	10	12	4					
	(19.5)	(100.0)		(22.2)	(26.7)	(8.9)	182	61	43	64	14
							(100.0)	(33.5)	(23.6)	(35.2)	(7.7)
**Takeaways**	873	128	44	25	31	28	416	130	74	138	74
	(39.0)	(100.0)	(34.4)	(19.5)	(24.2)	(21.9)	(100.0)	(31.3)	(17.8)	(33.2)	(17.8)
**National fast food chains**	33	1	0	1	0	0	7	1	1	3	2
	(1.5)	(100.0)	(0.0)	(100.0)	(0.0)	(0.0)	(100.0)	(14.3)	(14.3)	(42.9)	(28.6)
**Food stores**	735	84	28	19	21	16	346	99	75	107	65
	(32.9)	(100.0)	(33.3)	(22.6)	(25.0)	(19.0)	(100.0)	(28.6)	(21.7)	(30.9)	(18.8)
**Vans**	159	13	4	5	2	2	56	7	23	16	10
	(7.1)	(100.0)	(30.8)	(38.5)	(15.4)	(15.4)	(100.0)	(12.5)	(41.1)	(28.6)	(17.9)
**All**	2236	271	95	60	66	50	1007	298	216	328	165
	(100.0)	(100.0)	(35.1)	(22.1)	(24.4)	(18.5)	(100.0)	(29.6)	(21.4)	(32.6)	(16.4)

⁎ Deprivation scale (based on % of pupils eligible for free schools meals): Q1=lzeast deprived, Q4=most deprived.

**Table 2:** Ratio of the observed to expected *K*-function outlet densities at distances from Glasgow public secondary schools.

Distance from secondary schools				
	400 m	800 m	1200 m	1500 m
*Food outlet type*				
Cafés	2.92	2.79	2.40	2.34
Takeaway	3.84	2.96	2.44	2.28
National fast food chains	0.00⁎	1.44⁎	1.52⁎	1.64
Food stores	3.28	3.19	2.71	2.56
Vans	1.49⁎	1.91	1.79	1.87
All	3.45	3.07	2.60	2.48

*School deprivation quartile*All outlets				
Q1 (least deprived)	5.30	3.75	3.07	2.77
Q2	3.03	2.74	2.95	2.82
Q3	3.68	4.01	3.11	2.80
Q4 (most deprived)	1.99	1.94	1.44	1.66

⁎ No statistical evidence of clustering from assessment of plot with simulation envelope.

**Table 3:** Ratio of the observed to expected *K*-function outlet densities by food outlet type, stratified by deprivation.

	400 m	800 m	1200 m	1600 m
*Cafés*				
Q1 (least deprived)	5.77	3.66	2.91	2.41
Q2	2.40	2.52	2.80	2.77
Q3	2.64	3.90	2.94	2.82
Q4 (most deprived)	0.86⁎	1.03⁎	0.91⁎	1.31⁎

*Takeaway*				
Q1 (least deprived)	5.25	3.79	3.00	2.69
Q2	3.15	2.13	2.47	2.34
Q3	4.31	3.93	2.93	2.59
Q4 (most deprived)	2.68	2.01	1.40	1.53

*National fast food chains*				
Q1 (least deprived)	0.00⁎	1.30⁎	1.45⁎	1.30⁎
Q2	0.00⁎	0.65⁎	0.58⁎	1.30⁎
Q3	0.00⁎	1.34⁎	1.49⁎	1.52⁎
Q4 (most deprived)	0.00⁎	1.93⁎	2.00⁎	1.83⁎

*Food stores*				
Q1 (least deprived)	4.89	3.67	3.16	2.98
Q2	3.05	2.83	3.09	2.90
Q3	3.51	4.12	3.14	2.73
Q4 (most deprived)	1.87⁎	2.27	1.62	1.75

*Vans*				
Q1 (least deprived)	2.28⁎	1.14⁎	1.01⁎	1.05⁎
Q2	1.71⁎	3.84	2.91	2.71
Q3	1.17⁎	1.17⁎	1.81⁎	1.74⁎
Q4 (most deprived)	0.54⁎	1.07⁎	1.07⁎	1.56⁎

⁎ No statistical evidence of clustering from assessment of plot with simulation envelope.
